# The smallest Neoptera (Baryshnyalidae fam. n.) from Hagen-Vorhalle (early Late Carboniferous: Namurian B; Germany)

**DOI:** 10.3897/zookeys.130.1422

**Published:** 2011-09-24

**Authors:** Jan-Michael Ilger, Carsten Brauckmann

**Affiliations:** Clausthal University of Technology, Institute of Geology and Paleontology, Leibnizstraße 10, 38678 Clausthal-Zellerfeld, Germany

**Keywords:** Hagen-Vorhalle, Neoptera, *Baryshnyala occulta* gen. et sp. n., Namurian B, holometaboly

## Abstract

With *Baryshnyala occulta* **gen. et sp. n.** (Baryshnyalidae **fam. n.**) we report a new small species from early Late Carboniferous (Pennsylvanian) deposits in Hagen-Vorhalle. It differs in its unique venation pattern and small size from all other Neoptera known from this Lagerstätte and other contemporaneous locations worldwide. With an estimated wing length of <10 mm it is by far the smallest species of Neoptera from Hagen-Vorhalle and is less than half as long as *Heterologopsis ruhrensis* Brauckmann & Koch, 1982 (~25 mm). The specimen shows some relations to the earliest Holometabola and may date back the first appearance of holometaboly to the Namurian B (early Bashkirian: Marsdenian). The new species increases the paleo-biodiversity and span of inter-specific variability within the early Neoptera. It shows that very small and tiny specimens and species can easily be overlooked.

## Introduction

Hagen-Vorhalle is one of the most important Konservat-Lagerstätten of the early Late Carboniferous (Early Pennsylvanian) and has provided remarkable data of supra-regional importance about the evolution of the most ancient Pterygota.

An extraordinary insect fauna was discovered in 1982 and reported in a brief article by Brauckmann and Koch (1982). Over 16,000 samples were recovered in an excavation campaign in 1990–1997 and by private collectors. Insects are represented by ~310 individuals (mostly in dorsal view with part and counterpart). Hendricks (2005) published a complete list of determinable taxa whereas Brauckmann et al. (2010) presented an actual outline of the insect fauna and their relation to other locations. Most of the material is very well preserved, including basal Neoptera Martynov, 1923 with ~210 specimens in 5 species: *Kemperala hagenensis* Brauckmann, 1984, *Holasicia rasnitsyni* Brauckmann, 1984, *Kochopteron hoffmannorum* Brauckmann, 1984, *Heterologopsis ruhrensis* Brauckmann & Koch, 1982, and *Baryshnyala occulta* gen. et sp. n.

*Kemperala* Brauckmann, 1984 and *Holasicia* Kukalová, 1958 can be assigned to the family Paoliidae Handlirsch 1906. For diagnosis and included genera see Kukalová (1958), Brauckmann (1984), Brauckmann et al. (1985, 2003, 2010), Prokop and Nel (2007), Ilger and Brauckmann (2008), and Prokop et al. (in press). As shown in the historical compilation by Prokop and Nel (2007), the systematic rank of paoliids and related early Pterygota has been the subject of controversy during the last few decades: Carpenter (1992), for example, traditionally considered them as a family within the “Protorthoptera,” a clearly polyphyletic “order”. Kukalová-Peck and Brauckmann (1992) assigned the “paoliid line” together with other early Neoptera to the “hemipteroid stem assemblage”. Grimaldi and Engel (2005) placed them as Paoliidae directly in Neoptera without indication of any order. On the other hand, Rohdendorf and Rasnitsyn (1980) and subsequently Rasnitsyn and Quicke (2002) ranked them as Paoliida on ordinal level. Prokop and Nel (2007) did the same, and additionally introduced their Katerinkidae, a closely related new family of the same order. A more general critical discussion of different views of hexapod phylogeny including the Palaeoptera/early Neoptera problem has been provided by (Klass (2007, 2009). In the present contribution we avoid a precise higher systematic assignment, because we do not want to anticipate the final results of the current phylogenetic analyses.

The systematic position of the *Kochopteron* Brauckmann, 1984 and *Heterologopsis* Brauckmann & Koch, 1982—which are morphologically similar to the Paoliidae—is unclear and still under controversial discussion. Brauckmann et al. (2003) assigned them to the family ?Cacurgidae Handlirsch, 1911. In contrast, Béthoux and Nel (2002) placed *Heterologopsis* in the Archaeorthoptera.

*Baryshnyala occulta* gen. et sp. n. cannot be allocated to any of these groups and is a representative of a new family within the early Neoptera (Baryshnyalidae fam. n.)

## Methods

The specimen WMf.N P.21299 is stored in the Hagen-Vorhalle collection of the LWL-Museum für Naturkunde, Westfälisches Landesmuseum mit Planetarium in Münster. It was recovered in an excavation campaign in 1990–1991.

Investigations were done using a Euromex® ZT-45 zoom trinocular with an attached camera tube. Objects were illuminated with a Euromex® EK-1 cold light lamp with polarizing filters to minimize reflections on clay minerals and mica surfaces. Imaging conditions for photo documentation were optimized by varying illumination levels and angles with polarized and non-polarized light. Photographs were taken by using a Canon PowerShot A470 digital point-and-shoot camera with Super Macro function. Digital images were edited using the computer programs IMAGEJ, COREL PHOTO-PAINT and GIMP.

The nomenclature of wing venation follows Kukalová-Peck and Willmann (1990) as well as Kukalová-Peck and Brauckmann (1992). Main vein terminology corresponds to this scheme: ScP– = Subcosta posterior, RA+ = Radius anterior, RP– = Radius posterior, Ma+ = Media anterior, MP– = Media posterior, CuA+ = Cubitus anterior, CuP– = Cubitus posterior, AA+ = Analis anterior, arc = arculus (strong cross-vein between MP– and CuA+), cmf = cubito-median fold (nomenclature follows Nel et al. (2007): convex fold between MP– and CuA+). Attached plus and minus indicate the corrugation of the wing; i.e.: + = above plane of projection, – = below plane of projection.

## Systematics

### Infraclass Neoptera Martynov, 1923

#### Family Baryshnyalidae fam. n.

urn:lsid:zoobank.org:act:CBBFC06D-A915-4F58-8A9B-E9ED57BA9E71

http://species-id.net/wiki/Baryshnyalidae

##### Type (and only known) genus.

*Baryshnyala* gen. n., original designation.

##### Diagnosis.

 Wing small and well rounded, with the following venation pattern: (i) strong cross-vein between MP– and CuA+ in basal part of wing (arculus), (ii) pronounced convex fold between MP– and CuA+ (cubito-median fold), (iii) CuP– strongly convex, with 3 terminal branches, (iv) a number of straight cross-veins between main veins.

#### Baryshnyala gen. n.

urn:lsid:zoobank.org:act:B3D7979B-92A1-4ED8-B55C-A4858E47861B

http://species-id.net/wiki/Baryshnyala

##### Type (and only known) species.

*Baryshnyala occulta* gen. et sp. n., original designation.

##### Diagnosis.

 Very small (length <10 mm) and compact wing with well rounded apex. The venation pattern shows a combination of the following unique characters: (i) RP– branches far before reaching the mid-wing, (ii) well pronounced cubito-median fold, (iii) CuP1– very strongly convex, (iv) posterior branches of MP– and CuA+ curved backwards (strongly convex), (v) no archedictyon but a number of cross-veins mainly in distal half of the wing.

##### Remarks.

 Despite the small size of the wing the regular shape precludes that it is a nymphal wing as it shows no kind of a typical strong backward flexion.

##### Etymology.

 The genus name is a combination of the Russian “baryshnya“ for young, unmarried woman and Latin “ala” for wing; gender feminine.

#### Baryshnyala occulta gen. et sp. n.

urn:lsid:zoobank.org:act:A096877A-814A-4E30-B782-5E71ACF0FE5C

http://species-id.net/wiki/Baryshnyala_occulta

Fig 1A–B 2A–F

##### Holotype (and only known specimen).

 Specimen no. WMf.N P.212999, left metathoracic wing, with slightly damaged apex and lacking wing base. Stored in the collection of the LWL-Museum für Naturkunde, Westfälisches Landesmuseum mit Planetarium in Münster (Germany).

##### Type locality.

 Former brickyard quarry near Hagen-Vorhalle, North Rhine-Westphalia, Germany (topographic map 1 : 25,000 sheet no. 4610 Hagen/Westfalen; 51°22.88'N; 007°26.77'E, ~115 m a.s.l.).

##### Type stratum.

 Early Late Carboniferous (Early Pennsylvanian): early Bashkirian, late Namurian B, late Marsdenian, ammonoid zone R2c, Ziegelschiefer Formation.

##### Diagnosis.

 As for the genus (due to the temporarily monospecific status). Length ≥9.6 mm.

##### Preservation.

 The isolated wing is quite well preserved, though it lacks the proximal tenth with articulation and most of the anal area. Apical wing margin is also slightly damaged. The corrugation is easily discernible but flattened by diagenetic compression. The anterior midwing is slightly damaged by preparation marks which obscure the exact reconstruction of terminal ScP– and RA+ branches—especially whether they do or do not fuse.

Also visible in the apical area (Fig. 1), tiny prodissoconchs of young bivalves are attached to the dorsal wing membrane. These embryonic shells are common in basal Neoptera from Hagen-Vorhalle but are absent in all other Pterygota from the same locality. Most recently this has led to new interpretation of the taphonomy of the Lagerstätte.—A manuscript by Ilger is currently in review, Ilger and Brauckmann (2009) gave a short overview.—The model implies a stage of drifting along a pycnocline in a well stratified water body. During this period the corpses were affected by free-swimming bivalve larvae.

**Figure:**
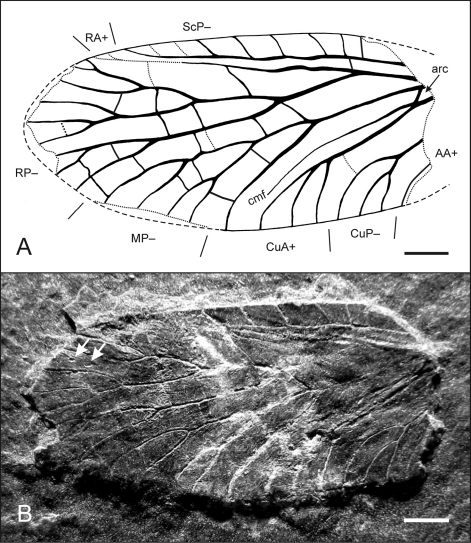
**Figure 1.** *Baryshnyala occulta* **gen. et sp. n.**; holotype, isolated left metathoracic wing (WMf.N P.21299); early Late Carboniferous (Early Pennsylvanian: Namurian B, Marsdenian): **A** superimposed drawing with interpretation of the wing venation pattern; black arrow indicates arculus (arc) **B** photograph under polarized light; white arrows indicate attached bivalve prodissoconchs. Scale bars: 1 mm.

##### Measurements.

 Preserved length: 8.7 mm; estimated length: ≥9.6 mm; maximum width (at most posterior branch of MP–): 4.2 mm. Approximated ratio length/width: ~2.3.

##### Description.

 There is only one isolated wing known. Due to its shape and the existence of a cubito-median fold we suppose it is a metathoracic wing (Figs 1–2). Costal margin in mid-wing straight, apex well rounded. Costal area strongly pectinate with wide-standing cross-veins. ScP– simple and most likely reaching costal margin. R stem proximally thick and strong, branching in proximal quarter of wing length. RA+ simple and generally straight, terminal development not well preserved but probably reaching costal margin in distal fifth. RP– well and strongly developed, with 9 terminal branches. MP– strong, with 5 terminal branches, first branching clearly before mid-wing and fanning at basal third of wing length, posterior branches with strongly curved backwards at posterior wing margin. Strong arculus (= cross-vein between MP– and CuA+) present in proximal eighth of the wing. CuA+ slightly reduced, slightly concave in the middle part before branching, with 3 terminal branches, all very strongly curved backwards. Well developed and probably sclerotizied cubito-median fold (between MP– and CuA+) running close to CuA+, especially in the proximal part. CuP– with 3 terminal branches, CuP1– very strongly convex. First AA+ straight. A number of thin and straight cross-veins mainly in the sectors of RP– and MP– and distal of mid-wing.

**Figure:**
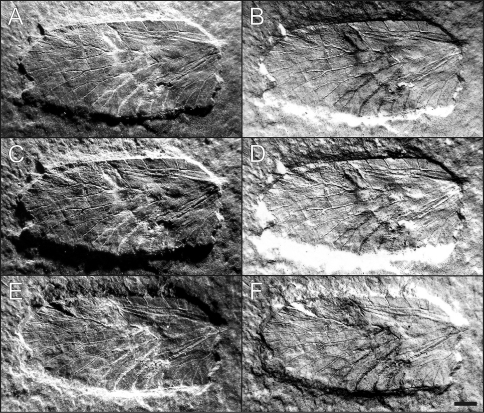
**Figure 2.** *Baryshnyala occulta* **gen. et sp. n.**; holotype, isolated left metathoracic wing (WMf.N P.21299); early Late Carboniferous (Early Pennsylvanian: Namurian B, Marsdenian): **A–F** The same wing in different illumination settings. Scale bar: 1 mm.

##### Etymology.

 Latin occultus, -a, -um (adjective) meaning arcane. The holotype specimen was stored for twenty years in the collection without being identified as a new species.

## Discussion

### Small basal Neoptera of the Late Carboniferous

*Baryshnyala occulta* gen. et sp. n. can be easily separated from all other Neoptera from Hagen-Vorhalle by its very small size (length: ≥9.6 mm, width: 4.2 mm). In comparison to other wings within this group the specimen is about half as long as *Heterologopsis ruhrensis* (length: ~25 mm, width: ~8 mm). *Holasicia rasnitsyni* (length: 36 mm, width: 13 mm) and *Kochopteron hoffmannorum* (length: 41 mm, width: 13 mm) are distinctly larger, and *Kemperala hagenensis* (length: 61 mm, width: 22 mm) is by far the largest species of basal Neoptera from Hagen-Vorhalle (Fig. 3). *Baryshnyala occulta* fits very well in this set of proportions: all species show a tendency towards a length/width ration about 3 (l/w = 2.8 in *Baryshnyala occulta*). *Kemperala hagenensis* and *Kochopteron hoffmannorum* seem to vary mostly in the wing length whereas the width is more consistent. In contrast, the wings of *Holasicia rasnitsyni* vary more in their width and less in length. The variation in *Holasicia ruhrensis* and *Baryshnyala occulta* is unknown because they are monotypic.

**Figure:**
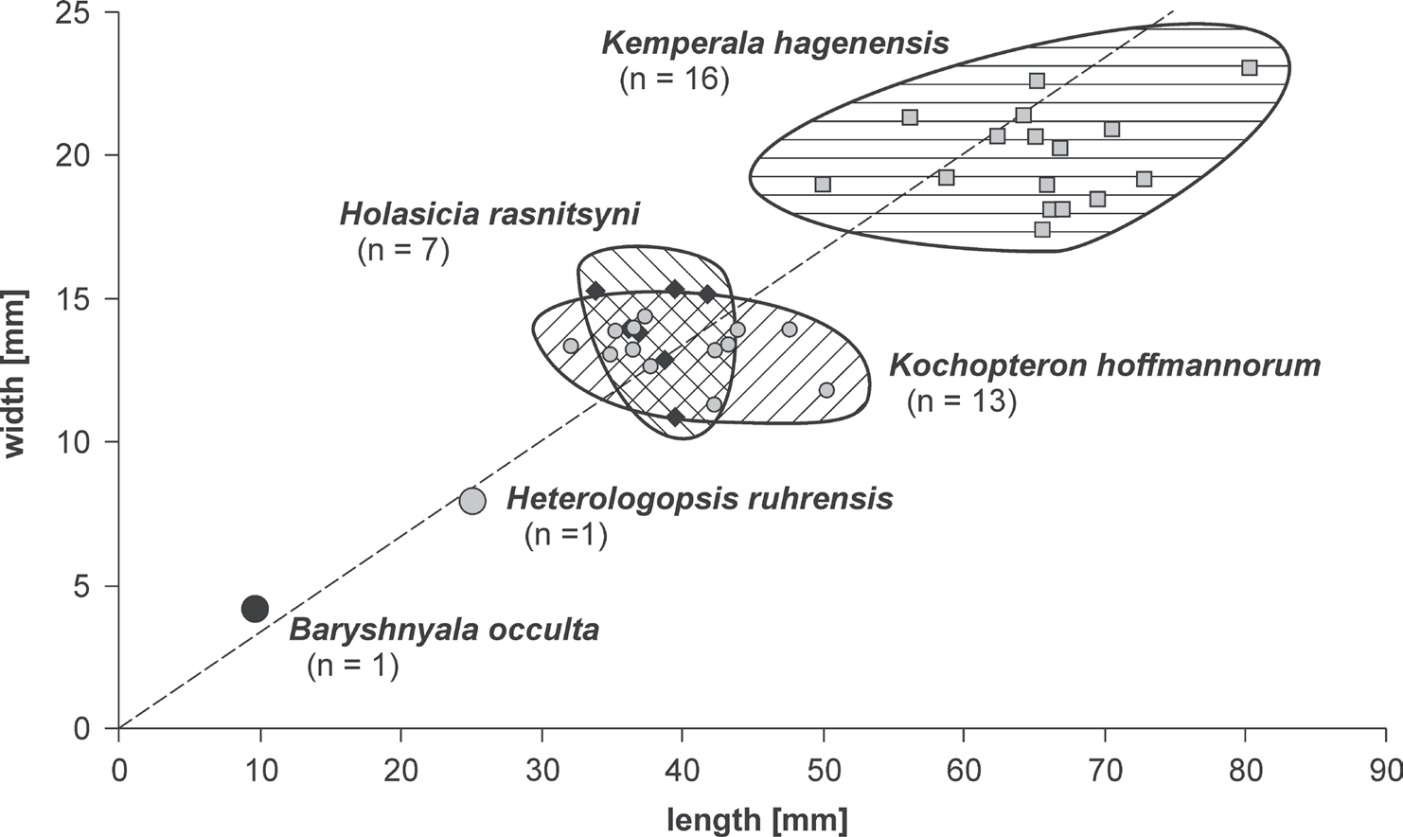
**Figure 3.** Synopsis of wing proportions in basal Neoptera from Hagen-Vorhalle. *Baryshnyala occulta* gen. et sp. n. fits very well in this set: all species show a tendency towards a length/width ration about 3 (dashed graph).

Further small Late Carboniferous (Pennsylvanian) basal Neoptera from localities in northwestern Germany and thus close to Hagen-Vorhalle are for example (arranged by their stratigraphical age):

*Metropatorites kassenbergensis* Keller, 1934 (ord. et fam. inc. sed.), Namurian C (Bashkirian: Yeadonian): preserved length: 9 mm, estimated total length: ~22 mm, preserved width: 5 mm, estimated maximal width: ~9 mm.

*Micropalentomum minusculum* Schmidt, 1962 (Micropalentomidae), late Westphalian A (Bashkirian: Langsettian): length: ~3,5 mm [sic!], width: ~1 mm.

*Eodelopterum priscum* Schmidt, 1962 (Miomoptera: Archaemiopteridae), early Westphalian B (late Bashkirian: Duckmantian): length: <5 mm, width: ~2.3 mm.

*Heterologellus teichmuellerae* Schmidt, 1962 (Protophasmatidae), early Westphalian C (Moskovian: Bolsovian): length: ~18 mm, width: ~7 mm.

*Controversala miomopteroides* Brauckmann & Herd, 2005 (ord. et fam. inc. sed.), Westphalian D (late Moskovian): preserved length: 19 mm, estimated total length: ~20 mm, width: 8 mm.

Worldwide there are known many other small Neoptera from the Late Carboniferous and Permian. Especially Early Permian Protomeropidae Tillyard, 1926 are very small. For example *Westphalomerope maryvonneae* Nel et al., 2007 from the early Langsettian (Late Carboniferous: Bashkirian, Westphalian A) of France is twice smaller than *Baryshnyala occulta*.

### Most ancient representative of Holometabola?

The probably earliest evidence for holometabolous insects is a metathoracic wing of *Westphalomerope maryvonneae*. As the authors correctly pointed out, Holometabola lack a distinct synapomorphy in their wing venation pattern. The allocation to Endopterygota Sharp, 1898 is done by attribution of *Westphalomerope maryvonneae* to a subgroup of this clade, e.g. the family Protomeropidae. An adapted diagnosis of the family is given by Sukatsheva (1976). The two main characters are (terminology changed to the scheme used in the present paper): (i) a brace (= arculus) between CuA+ and MP–, and (ii) a heavily sclerotizied convex fold in front of CuA+ (= cubito-median fold). Such fold is not restricted to Holometabola but can also be found e.g. in *Evenka archaica* Rasnitsyn, 1977 which is probably close related to Paoliidae. Furthermore an arculus is also widespread and can be found in Paoliidae and other groups as well.

Both species, *Baryshnyala occulta* gen. et sp. n. and *Westphalomerope maryvonneae*, share the following characters within the diagnosis by Kukalová-Peck and Willmann (1990; terminology here adapted): (i) narrow costal area, (ii) very narrow area between ScP– and RA+, (iii) arculus present (typical for Permian Protomeropidae but lacking in *Westphalomerope maryvonneae*), (iv) well developed and prominent cubito-median fold, sub-parallel to CuA(1)+, (v) branching of Cu close to wing base (this part of the wing is not preserved in *Baryshnyala occulta* but can be extrapolated from the course of the preserved parts of CuA+ and CuP–), (vi) branched CuA+ (probably plesiomorphic).

*Westphalomerope maryvonneae* is quite similar to other hindwings in Protomeropidae. *Baryshnyala occulta* however differs from all Protomeropidae in the following characters: (i) ScP– reaching anterior wing margin and probably distally not fused with RA+, (ii) CuA– not simple but with 3 terminal branches, and—probably most important character—(iii) CuP– branched as rich as CuA+, the first branch (CuP1–) extremely curved backwards. The first character is regarded as an apomorphy of the Protomeropidae by Ivanov and Sukatsheva (2002), whereas Nel et al. (2007) assume it to be a plesiomorphy at the level of Holometabola or even the whole Pterygota. Other plesiomorphic characters of *Baryshnyala occulta* are the rich branching of ScP– (with 8 anterior branches) and RP– (with 9 terminal branches). The rich branching of CuP– and backward-curving especially of CuP1– is quite unique in Late Carboniferous basal Neoptera.

Therefore we conclude that *Baryshnyala occulta* is vague related to the Protomeropidae but represents a separate new family of uncertain systematic placement. Both taxa might be stem group representatives of Endopterygota. In this case *Baryshnyala occulta* would be the most ancient holometabolous insect.

## Conclusions

The previously known species of Neoptera from Hagen-Vorhalle are of medium to rather large size. *Baryshnyala occulta* gen. et sp. n. shows that expanded and carefully directed prospection methods will increase the chance of also finding small insects. A similar conclusion is reached by Zessin et al. (2011).

Very small species and specimens are always in danger of being overlooked during their excavation and subsequent scientific handling. *Baryshnyala occulta* gen. et sp. n. obviously differs from all other Neoptera and “Palaeoptera” species known from Hagen-Vorhalle by its small size and venation pattern.

Similarities in the venation pattern and the occurrence of a well developed cubito-median fold indicate a systematic position distantly related to the family Protomeropidae but possibly within the Endopterygota. If so, this would date the first occurrence of holometaboly back to the Namurian B (early Bashkirian: Marsdenian).

*Baryshnyala occulta* shows that the early Neoptera in general can be expected to be much more frequent and diverse than currently known. It is the fifth Neoptera species from this important paleo-ecosystem and enlarges the morphological range of this group. Up to now small Late Carboniferous Pterygota are generally rare, and each new specimen can be essentially important for the knowledge of their early evolution. Further examinations, even in already stored collections, are expected to yield surprising discoveries.

## Supplementary Material

XML Treatment for Baryshnyalidae

XML Treatment for Baryshnyala

XML Treatment for Baryshnyala occulta
